# Hand-held cardiac ultrasound examinations performed in primary care patients by nonexperts to identify reduced ejection fraction

**DOI:** 10.1186/s12909-019-1713-9

**Published:** 2019-07-25

**Authors:** G. Nilsson, L. Söderström, K. Alverlind, E. Samuelsson, T. Mooe

**Affiliations:** 1 Department of Public Health and Clinical Medicine, Umeå University, 90187 Umeå, Sweden; 2 Department of Public Health and Clinical Medicine, Unit of Research, Education and Development-Östersund Hospital, Box 654, 83127 Östersund, Sweden; 3 Unit of Research, Education and Development-Östersund Hospital, Region Jämtland Härjedalen, Östersund, Sweden

**Keywords:** Echocardiography, Heart failure, Primary care, Clinical trial

## Abstract

**Background:**

Early identification of patients with reduced left ventricular ejection fraction (LVEF) could facilitate the care of patients with suspected heart failure (HF). We examined if (1) focused cardiac ultrasound (FCU) performed with a hand-held device (Vscan 1.2) could identify patients with LVEF < 50%, and (2) the distribution of HF types among patients with suspected HF seen at primary care clinics.

**Methods:**

FCU performed by general practitioners (GPs)/GP registrars after a training programme comprising 20 supervised FCU examinations were compared with the corresponding results from conventional cardiac ultrasound by specialists. The agreement between groups of estimated LVEF < 50%, after visual assessment of global left ventricular function, was compared**.** Types of HF were determined according to the outcomes from the reference examinations and serum levels of natriuretic peptides (NT-proBNP).

**Results:**

One hundred patients were examined by FCU that was performed by 1–4 independent examiners as well as by the reference method, contributing to 140 examinations (false positive rate, 19.0%; false negative rate, 52.6%; sensitivity, 47.4% [95% confidence interval [CI]: 27.3–68.3]; specificity, 81.0% [95% CI: 73.1–87.0]; Cohen’s κ measure for agreement = 0.22 [95% CI: 0.03–0.40]). Among patients with false negative examinations, 1/7 had HF with LVEF < 40%, while the others had HF with LVEF 40–49% or did not meet the full criteria for HF. In patients with NT-proBNP > 125 ng/L and fulfilling the criteria for HF (68/94), HF with preserved LVEF (≥50%) predominated, followed by mid-range (40–49%) or reduced LVEF (< 40%) HF types (53.2, 11.7 and 7.4%, respectively).

**Conclusions:**

There was poor agreement between expert examiners using standard ultrasound equipment and non-experts using a handheld ultrasound device to identify patients with reduced LVEF. Asides from possible shortcomings of the training programme, the poor performance of non-experts could be explained by their limited experience in identifying left ventricular dysfunction because of the low percentage of patients with HF and reduced ejection fraction seen in the primary care setting.

**Trial registration:**

The study was registered at ClinicalTrials.gov (NCT02939157). Registered 19 October 2016.

## Background

Patients attending primary care with symptoms indicating heart failure (HF) could benefit from faster diagnostic procedures that are conducted at the point of care. After clinical evaluation and electrocardiography, the next step in diagnosing HF is normally testing for natriuretic peptides and an echocardiography, which are performed at a hospital clinic. Because no single symptom of HF is specific, echocardiography is mandatory for establishing the diagnosis and to distinguish between the different types of HF. Additionally, it has important implications for the therapeutic possibilities [1–6]. The terminology of HF has been revised and three subtypes of HF are described based on levels of natriuretic peptides and left ventricular ejection fraction (LVEF); HF with reduced ejection fraction (HFrEF), HF with mid-range ejection fraction (HFmrEF), and HF with preserved ejection fraction (HFpEF) [6]. Since serum levels of natriuretic peptides overlap between the types of HF, and elevated values are also seen in patients with other medical conditions (e.g., renal failure, atrial fibrillation, and advanced age), natriuretic peptide levels are mainly used to rule-out HF in patients with a level below the cut-point for exclusion (N-terminal pro-B–type natriuretic peptide [NT-proBNP] < 125 pg/ml in the non-acute setting) [7–14]. In recent years, examinations performed by handheld ultrasound platforms were introduced as an extension of the traditional clinical examination of patients presenting with cardiac symptoms, an examination known as focused cardiac ultrasound (FCU). FCU performed with handheld devices has limitations demanding an evaluation in the clinical setting where the technique is intended for use [15–18]. These handheld ultrasound devices provide a two-dimensional view of the heart, and some also have a colour-Doppler mode but with no continuous or pulsed Doppler modes, which limits the range of possibilities for diagnosing diastolic dysfunction [19–22]. The possibility of diagnosing left ventricular systolic impairment with good accuracy using hand-held ultrasound devices is reported in several studies that were conducted at cardiology wards and in other hospital settings [21–26]. Early identification of reduced LVEF in patients with symptoms indicative of HF could facilitate the care of patients that are evaluated by general practitioners (GPs). However, only a few studies of FCU have been reported so far from relevant primary care settings [27, 28]. Therefore, we examined whether FCU could be used to identify patients with reduced ejection fraction (LVEF < 50%) among patients with suspected HF visiting primary care clinics. Furthermore, we examined the distribution of HF classes, in the population studied.

## Methods

### Design

FCU was conducted by non-expert physicians after a training programme comprising 20 supervised sessions. Conventional cardiac ultrasound was performed by specialized staff as a reference.

### Setting and participants

Men and women aged ≥20 years residing in the Region Jämtland Härjedalen, northern Sweden (adult population 100,396 inhabitants at the end of 2016) were eligible. Primary-care patients who were referred for ultrasound examinations to diagnose or to guide treatment of HF were invited to the study. The FCU examinations were performed at the Clinical Research Centre and reference examinations were performed at the Department of Clinical Physiology, both at Östersund Hospital. Enrolment was carried out from December 12, 2016 to June 15, 2017. Patients referred for follow-up of cardiac valve disorders, without a question of HF, were excluded.

Five GP registrars and one GP with no prior experience in cardiac ultrasound participated as examiners using FCU. Each patient in the study could be examined during the same visit by 1–4 examiners independently from each other. This design was chosen to enable more study examinations compared to a 1:1 design. The study and reference examinations were scheduled on the same day, with study examinations performed before the reference examinations whenever possible. The examiners evaluated their findings independently immediately after the examination and recorded the results in the patient’s examination protocol.

### Training programme

Primarily, the participating examiners received a 2-h lecture about the principles of diagnostic ultrasound and demonstrations of FCU and comprehensive cardiac ultrasound at the medical ward, Levanger Hospital, Norway. Thereafter, all subsequent training was conducted at the Clinical Research Centre, Östersund Hospital. The examiners received a textbook, and they were instructed to study the background of cardiac ultrasound and the video loops that showed normal and impaired cardiac function, which were provided in the corresponding e-book [29]. The examiners were also instructed to study video tutorials of cardiac ultrasound, which could be accessed on the website of the University of South Carolina School of Medicine [30]. A qualified ultrasound technician supervised the training. All six of the examiners in the study performed the twenty FCU examinations stipulated on individual study patients under supervision. After that, later examinations were performed by the examiners independently. The supervised training sessions were scheduled every other week from December 12, 2016, with the last examiner completing the training period on April 18, 2017. The training focused on obtaining representative imaging views for assessment of cardiac function in defined standard-imaging views (parasternal long-axis and short-axis views, apical 4-chamber and subcostal views). LVEF was evaluated through visual assessment of global left ventricular function and graded as normal (≥50%), reduced (< 50%), or severely reduced (< 30%). After examining the last study patient enrolled in the study, the recorded film sequences were evaluated for quality by a cardiologist experienced in cardiac ultrasound, without access to any other patient-related data. The examinations were anonymised with respect to the identity of the examiner and study patient and evaluated in random order as “acceptable” (1) or “not acceptable” (0) for diagnostic purposes. Inadequate projections and failure to record images were classified as “not acceptable” (0).

### Study and reference examinations

The FCU examinations (study method) were performed with the imaging device, Vscan V1.2 (GE Vingmed Ultrasound, Horten, Norway, CE0470). The Vscan is equipped with a phased array transducer (1.7–3.8 MHz), and has a screen dimension of 8.9 cm, image resolution (pixels) of 240 × 320, and grey scale and colour Doppler. The Vscan platform allows for digital storage of still frames and loops of cardiac cycles predefined to 2 s without ECG signal, M-Mode, and continuous or pulsed Doppler modalities [19, 31]. The recordings were stored on a micro-SD card and transferred using commercial software (Gateway; GE Vingmed Ultrasound) to a separate computer. The reference examinations were conducted on a Siemens Acuson S2000 platform including two-dimensional Doppler and tissue Doppler modalities for assessing systolic and diastolic functions. LVEF was assessed visually and graded as markedly reduced (< 30%), reduced (< 40%), mid-range (40–49%), or preserved (≥50%). Diastolic function was evaluated according to Doppler estimates of velocities and deceleration times [32]. The reference examinations were conducted by qualified ultrasound technicians and evaluated by physicians specialized in clinical physiology or cardiology. Results of the reference and study examinations were not communicated to study patients during the examination sessions. A notice stating the results of the reference examination was sent back to the patient’s GP. Observations from the FCU examinations were only used within the study.

The examinations were conducted in the left lateral position (parasternal and apical views) or in the supine position (subcostal view). The duration of the study examinations was about 15 min, excluding time for protocol-related procedures.

When each study examination was completed, serum NT-proBNP levels were analysed on a Cobas 6000 M-module (Roche Diagnostics), with a range of measurement of 5–35,000 ng/L (Department of Clinical Chemistry, Östersund Hospital).

### Outcome measurements

Agreement between the FCU and reference method was estimated, with a cut-off at LVEF < 50%. Heart failure criteria and classification were based on the results from the reference examinations and NT-proBNP levels. HF in study patients was classified according to the 2016 ESC Guidelines [6]: “Heart failure with reduced ejection fraction (HFrEF): Patients with LVEF <40%. Heart failure with mid-range ejection fraction (HFmrEF): Patients with LVEF 40% to 49%, NT-proBNP > 125 ng/L, and at least one additional criterion: Signs of relevant structural heart disease (LVH and/or LAE), or diastolic dysfunction. Heart failure with preserved ejection fraction (HFpEF): Patients with LVEF ≥50%, NT-proBNP > 125 ng/L, and at least one additional criterion: Signs of relevant structural heart disease (LVH and/or LAE), or diastolic dysfunction, (LVH= left ventricular hypertrophy; LAE= left atrial enlargement; diastolic dysfunction = assessment through conventional ultrasound examination incorporating relevant two-dimensional and Doppler data)”.

#### Study size

The study was approved to include up to 250 study patients (including patients examined during the training period). The sample size was estimated from a pragmatic standpoint, based on the availability of study patients, time, and funding constraints and previously published experiences [28].

#### Data analysis

Demographic data are presented as proportions, means ± standard deviations, or median and interquartile range for data not following a normal distribution. Between-group analysis of proportions was made via *Χ*^2^ statistics or the Fisher exact test, as applicable. Agreement between the study and reference methods (LVEF < 50%) were calculated via the Cohen’s kappa coefficient (*κ*)*,* and sensitivity and specificity were determined from the proportion of patients with true-positive and true-negative results, with 95% confidence intervals (CIs). Sensitivity and specificity calculations with 95% CIs were calculated with the software application WINPEPI, version 11.26 [33]. Other statistical analyses were performed with IBM SPSS (version 23).

## Results

Of 282 eligible study patients, 158 were enrolled, of which 58 patients were only examined during the training period. Enrolment was stopped after 6 months due to the time constraints of the study plan. The mean age of study patients was 69.9 years. Limited physical ability (slight limitation 37.8%, marked limitation 18.6%), exertional chest pain (32.3%), and cardiovascular and pulmonary comorbidities (hypertension 61.4%, previous myocardial infarction 10.2%) were common (Table 1).

**Table 1:** Characteristics of the study patient participants (*n* = 158)

Age, mean (SD), years	69.9 (11.9)
Female sex	71 (44.9%)
NT-proBNP, ng/L, median (IQR)	195 (738)
Body mass index in kg/m^2^, mean (SD)	28.2 (4.2)
Medical history, n/N (%)	
Chronic Heart failure, medication for	34/158 (21.5%)
Hypertension, medication for	97/158 (61.4%)
Diabetes mellitus, treatment for	28/158 (17.7%)
Dyslipidaemia, medication for	55/158 (34.8%)
Asthma or COPD	24/158 (15.2%)
Revascularisation	21/158 (13.3%)
AMI	16/157 (10.2%)
Stroke or TIA	18/158 (11.4%)
Symptoms, n/N (%)	
Orthopnea	23/157 (14.6%)
Nocturnal dyspnoea	30/158 (19.0%)
Ankle oedema	50/158 (31.6%)
Limitation of physical activity (NYHA I-IV), n/N (%)	
No limitation of physical activity	65/156 (41.7%)
Slight limitation of physical activity	59/156 (37.8%)
Marked limitation of physical activity	29/156 (18.6%)
Discomfort with any physical activity/ symptoms occurring even at rest	3/156 (1.9%)
Exertional chest pain, n/N (%)	50/155 (32.3%)

*NT-proBNP* N-terminal pro-B-type natriuretic peptide, *COPD* Chronic obstructive pulmonary disease, revascularisation = coronary bypass grafting or percutaneous coronary intervention; *AMI* Acute myocardial infarction, *TIA* Transitory ischaemic attack, *NYHA* New York Heart Association Functional Classification, *SD* Standard deviation, *IQR* Interquartile range

**Fig. 1:**
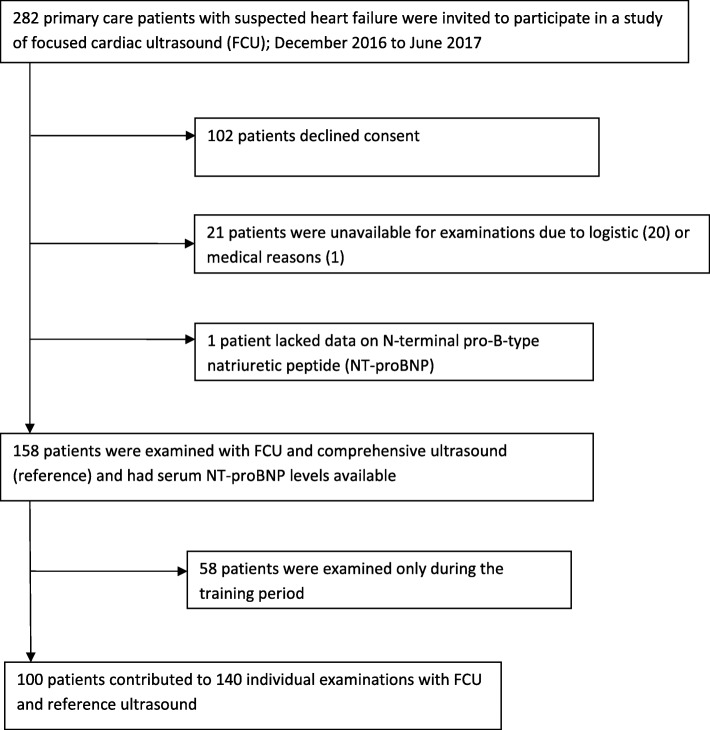
Study profile of patient recruitment

One hundred individual patients were examined with both FCU and the reference method, contributing to 140 individual study examinations (Fig. 1). Of the study patients, 65 were examined by 1 examiner, 31 by 2 examiners, 3 by 3 examiners, and 1 patient by 4 examiners. Each examination was performed independently of the others. The number of independent examinations per examiner after the training period was 7–76, (median 15) (Table 2). One hundred and eleven patients were examined with FCU before and 47 after the reference examination, with an overall median time difference of 1.5 h. During the training period, 80.0% of pictures obtained in the parasternal and apical views were evaluated as having acceptable image quality for diagnostic purposes, and the corresponding proportion in the independently obtained pictures was 80.6%. The proportion of images that were of acceptable quality in the subcostal view was lower; overall, it was 39.8%.

**Table 2:** Number of independently performed study examinations per examiner after training period

Examiner Number	Number of examinations after the training period
1	76
2	20
3	19
4	10
5	8
6	7
Total	140

Agreement between the FCU and reference methods in identifying LVEF < 50% were as follows: false positive rate, 19.0%; false negative rate, 52.6%; sensitivity, 47.4% (95% CI 27.3–68.3); specificity, 81.0% (95% CI 73.1–87.0); and Cohen’s κ value, 0.22 (95% CI 0.03–0.40) (Table 3). In patients with NT-proBNP-values > 125 ng/L, the agreement between the study and reference methods remained low (Cohen’s κ = 0.26 [95% CI 0.03–0.48]), false positive rate 22.9%, false negative rate 47.1%). Among the 7 individual study patients with a false negative examination (LVEF < 50% by reference examination but not by FCU), 1 patient fulfilled the criteria for HFrEF, 4 patients fulfilled criteria for HFmrEF, and 2 patients did not meet the defined criteria for HF according to the reference examination and NT-proBNP levels (10 examinations conducted in 7 patients). Among study patients with a false positive examination (LVEF < 50% by FCU but not by reference), 12/21 fulfilled the criteria for HFpEF (23 examinations conducted in 21 study patients). Because few patients had LVEF < 30%, these patients’ data were not treated separately in the analyses.

**Table 3:** Left ventricular ejection fraction (LVEF) determined by focused cardiac ultrasound (FCU) versus the reference examination^a^

		Assessment of LVEF by FCU		
		LVEF < 50%	LVEF ≥ 50%	Total
Comprehensive ultrasound (reference)	LVEF < 50%	9 (47.4%)	10 (52.6%)	19 (13.6%)
	LVEF ≥ 50%	23 (19.0%)	98 (81.0%)	121 (86.4%)
	Total	32 (22.9%)	108 (77.1%)	140

^a^A total of 140 FCU examinations were performed in 100 patients

The concordance between FCU and the reference method showed no trend toward an increase in the number of examinations per examiner (*p*-value for trend = 0.298) (Table 4). Among the six FCU examiners, the concordance between independently performed FCU examinations and the reference method ranged between 55.0 and 87.5% (mean 76.4%).

**Table 4:** Agreement (LVEF < 50%) between focused cardiac ultrasound (FCU) and comprehensive ultrasound (reference)^a^

Number of examinations	Examination result		Total number of examinations
	Concordance	Discordance	
1–10	19 (76.0%)	6 (24.0%)	25
11–20	26 (66.7%)	13 (33.3%)	39
> 20	62 (81.6%)	14 (18.4%)	76
Total	107 (76.4%)	33 (23.6%)	140

^a^The concordance or discordance by the number of FCU examinations independently performed per examiner in 100 study patients after an initial training period were compared with the reference method. Concordance was the agreement on the assessment of LVEF (< 50% cut-off) by both methods

*LVEF* Left ventricular ejection fraction

The NT-proBNP levels (range 5 to 9923 ng/L) overlapped between patients with and without HF, but with a lower median value among patients without HF criteria (median 65 ng/L; range 5 to 1292). All patients diagnosed with HFrEF had a NT-proBNP level that exceeded 700 ng/L (Table 5). In patients with a NT-proBNP value > 125 ng/L, HF criteria (HFpEF, HFmrEF or HFrEF) were fulfilled in 68/94 (72.3%); 50 for HFpEF (53.2%), 11 for HFmrEF (11.7%), and 7 for HFrEF (7.4%). No patient with a BNP-level ≤ 125 ng/L (*n* = 64) had HF (Table 6).

**Table 5:** Heart failure types and their relationship with natriuretic peptide (NT-proBNP) levels in primary care patients^a^

Heart failure type	NT-proBNP (ng/L), median (min - max)	Number of patients (%)
Heart failure with preserved ejection fraction (HFpEF)	757 (131–9923)	50 (31.6%)
Heart failure with mid-range ejection fraction (HFmrEF)	1311 (239–2656)	11 (7.0%)
Heart failure with reduced ejection fraction (HFrEF)	931 (709–5595)	7 (4.4%)
Heart-failure criteria not fulfilled	65 (5–1292)	90 (57.0%)

^a^Patients were examined with comprehensive cardiac ultrasound (*n* = 158)

*NT-proBNP* N-terminal pro-B type natriuretic peptide, serum level in ng/L. HFpEF was defined as LVEF ≥50%, NT-proBNP > 125 ng/L, and at least one additional criterion; a) signs of relevant structural heart disease (LVH and/or LAE) or b) diastolic dysfunction. HFmrEF was defined as LVEF 40 to 49%, NT-proBNP > 125 ng/L, and at least one additional criterion; a) or b). HFrEF was defined as LVEF <40%. *LVH* Left ventricular hypertrophy, *LAE* Left atrial enlargement

**Table 6:** Diagnostic outcomes by NT-proBNP (125 ng/L cut-off)

Diagnostic outcome	NT-proBNP > 125 ng/L (*n* = 94), n (%)	NT-proBNP ≤125 ng/L (*n* = 64), n (%)	P value for difference
HFpEF	50 (53.2%)	NA	NA
HFmrEF	11 (11.7%)	NA	NA
HFrEF	7 (7.4%)	0	0.042
Heart-failure criteria not fulfilled	26 (27.7%)	64 (100%)	< 0.001

*NT-proBNP* N-terminal pro-B type natriuretic peptide, serum level in ng/L. HFpEF was defined as LVEF ≥50%, NT-proBNP > 125 ng/L, and at least one additional criterion; a) signs of relevant structural heart disease (LVH and/or LAE) or b) diastolic dysfunction. HFmrEF was defined as LVEF 40 to 49%, NT-proBNP > 125 ng/L, and at least one additional criterion; a) or b). HFrEF was defined as LVEF <40%. *LVH* Left ventricular hypertrophy, *LAE* Left atrial enlargement. Patients were treated in a primary care setting and examined by comprehensive cardiac ultrasound (*n* = 158)

## Discussion

This clinical trial showed that FCUs performed by GPs in the primary care setting failed to identify patients with impaired LVEF, when a comprehensive cardiac ultrasound was used as the reference. GPs attended a training programme comprising 20 supervised FCU sessions before the start of the study. However, the agreement between FCU and comprehensive cardiac ultrasound by experts (reference) was low (Cohen’s κ value = 0.22; sensitivity 47.4%; specificity 81.0%). Of patients with a false negative result, only one had HFrEF (LVEF< 40%), while the other patients with false negative results had HFmrEF (LVEF 40–49%) or did not fulfil the criteria for HF according to the 2016 ESC guidelines. In patients with NT-proBNP > 125 ng/L, the levels of NT-proBNP did not differentiate between the types of HF (HFrEF, HFmrEF, HFpEF), with HFpEF as the predominant type, before HFmrEF and HFrEF. No HF was diagnosed in patients with NT-proBNP levels ≤125 ng/L.

In this study, the poor agreement between FCU and comprehensive cardiac ultrasound conflicts with previous reports of FCU conducted by both expert [21, 23, 31] and non-expert examiners that found a good overall agreement in patients recruited from hospital-based medical wards [24, 25, 34–36], with a diagnostic accuracy greater than 90% in some reports. The reasons for the disagreement between findings might include the following:

1. The supervised FCU training sessions were mainly focused on acquiring representative imaging views and less focused on interpretation of findings.
2. The use of web-based tutorials provided no opportunity for feedback on the cardiac function assessments during individual examinations. Mjölstad and colleagues reported a sensitivity of 92% and specificity of 94% using a hand-held ultrasound device (Vscan 1.2) and eye-balling of the LVEF as “>45, 30-45, or <30%, corresponding to normal/near normal, moderate, or severe dysfunction, respectively”. In this study, the participating residents had a personal supervisor with whom they could discuss their findings during a tutorial period comprising at least 100 examinations [24].
3. The number of supervised training sessions and the period for learning on the FCU might have been insufficient to learn the technical aspects of FCU and to gain confidence in interpreting the images. Nonetheless, the amount of training in the prior positive reports of FCU learning programmes for non-experts was highly diverse, ranging from 2 h to 3 months [24, 25, 34–39] or 10 to 100 examinations [26, 40–42].
4. Differences in the types of outcome measurements; in previous reports on training programmes for handheld ultrasound devices designed for GP graduates or GP registrars (training periods ranging from 8 h to 4 weeks), success was assessed by proxy measurements. These measurements included septal mitral annular excursion (sMAE), a surrogate measure of left ventricular systolic function, improvement on examiner’s test-scores, or self-perceived proficiency [28, 43, 44]. Even when accounting for differences in outcome measurements, there is no consensus concerning the ideal training programme for use of FCU by non-experts. The design of our training programme, with 20 supervised examinations, focused on technique and web-based learning about the interpretation of images, and was based on a pragmatic point of view after a search of the relevant literature.
5. The demography and types of HF differ between patients seen in cardiology wards and in primary care clinics, with more patients with HFpEF, or “diastolic heart failure”, seen in the primary care setting [45]. This has consequences due to the technical limitations of the handheld ultrasound devices (Vscan and other models), since evaluation of diastolic dysfunction demands Doppler modes that are not provided in the handheld platforms [19, 20]. In study patients with a false negative examination (LVEF< 50% by reference but not by FCU), one of seven patients was type HFrEF according to the reference examination, while the other patients were type HFmrEF or did not fulfil the complete criteria for HF. Thus, the poor performance in acquiring and assessing FCUs shown by the participants in our study could be linked to the low prevalence of patients with HFrEF, relative to the numbers of patients with the other types of HF commonly observed in the primary care setting. Since the symptoms of HF are nonspecific and not discriminatory between the types of HF, the issue of diagnosing all types of HF correctly is still essential due to differences in prognoses and therapeutic options; e.g., reductions in morbidity and mortality from pharmacotherapy are only shown in patients with LVEF reduced < 40% [6, 46, 47].

The serum NT-proBNP levels between types of HF, and between patients with and without HF, overlapped, although the median value was higher in patients who fulfilled the HF criteria. In patients with NT-proBNP > 125 ng/L, the poor agreement in LVEF between FCU and comprehensive cardiac ultrasound remained, indicating that pre-selection of patients by NT-proBNP levels > 125 ng/L will not necessarily lead to more accurate diagnostic results, although patients with NT-proBNP levels ≤125 ng/L are highly unlikely to have HF. The predominance of HFpEF before patients with HF with mid-range or reduced EF and the high prevalence of hypertension was in line with previous reports from population-based cohorts [45, 48, 49].

The overall percentage of ultrasound images that were of acceptable quality (about 80%) obtained in the main imaging views (parasternal long- and short-axis and apical four chamber) was comparable to those in previous studies (73.8–89%) [27, 34, 37]. In our study, we found that the subcostal imaging view was the most difficult to obtain correctly, similar to findings reported by Kobal et al. [37]. We would have preferred to provide prolonged training, with feedback for each trainee on their own FCU examinations; however, limited access to appropriately trained supervisors is a barrier to expanding such training programmes in the primary care setting [50]. Adequate image quality does not necessarily correspond to a correct assessment of cardiac function. Thus, sending the FCUs to a remote expert for interpretation might be a solution, particularly in remote areas [27, 51]. Since the agreement between the FCU and comprehensive cardiac ultrasound results was low, our training programme should be modified; e.g., with more opportunities to receive feedback on interpretation of the images. The ideal FCU training programme remains to be determined.

Our study has limitations. Only six individual examiners were evaluated in the training programme. The demographics of the non-consenting patients, about one third of all those eligible, are unknown, but could have influenced the results. The reference examinations were conducted by different expert examiners following a protocol for cardiac ultrasound examination in patients under normal care.

In further research on FCU in a primary care setting, remote expert interpretative support and methods to overcome the difficulties in assessing patients with HF who have mid-range and preserved EF should be addressed.

## Conclusions

There was poor agreement between findings from conventional ultrasound equipment and those from a handheld device used by non-experts in identifying reduced LVEF. Besides the limitations in the number of supervised training sessions and feedback opportunities, the poor performance of FCU in our study could be explained by the criterion chosen for reduced LVEF and a lower prevalence of patients with reduced LVEF in the primary care setting.

## Data Availability

The datasets obtained during the study will be available from the corresponding author on reasonable request.

## Abbreviations

CI: Confidence interval
FCU: Focused cardiac ultrasound
GP: General practitioner
HF: Heart failure
HFmrEF: Heart failure with mid-range ejection fraction
HFpEF: Heart failure with preserved ejection fraction
HFrEF: Heart failure with reduced ejection fraction
LAE: Left atrial enlargement
LVEF: Left ventricular ejection fraction
LVH: Left ventricular hypertrophy
NT-proBNP: N-terminal pro-B–type natriuretic peptide
sMAE: Septal mitral annular excursion
κ: Cohen’s kappa coefficient
